# Intuition is useful for medical practitioners but it cannot replace methodological knowledge in medical research: a case of ordered categorical outcomes

**DOI:** 10.3325/cmj.2020.61.469

**Published:** 2020-10

**Authors:** Vladimir Trkulja, Pero Hrabač

**Affiliations:** 1 Department of Pharmacology, Zagreb University School of Medicine, Zagreb, Croatia *vtrkulja@mef.hr*; 2 Department of Medical Statistics, Epidemiology, and Medical Informatics, “Andrija Štampar” School of Public Health, University of Zagreb School of Medicine, Zagreb, Croatia

In 1994, the *British Medical Journal* published an editorial entitled “The Scandal of Poor Medical Research” (1). The word “scandal” might sound harsh, but the text was motivating, not offensive. It primarily addressed research involving humans conducted (and read) by medical doctors. Instead of re-telling the editorial, we will illustrate its main points by a few quotes (1):

“*What should we think about a doctor who uses the wrong treatment, either wilfully or through ignorance, or who uses the right treatment wrongly (such as by giving the wrong dose of a drug)? Most people would agree that such behaviour was unprofessional, arguably unethical, and certainly unacceptable.*

*What, then, should we think about researchers who use the wrong techniques (either wilfully or in ignorance), use the right techniques wrongly, misinterpret their results, report their results selectively, cite the literature selectively, and draw unjustified conclusions? We should be appalled. Yet numerous studies of the medical literature, in both general and specialist journals, have shown that all of the above phenomena are common.^1 2 3 4 5 6 7^* …..*Why are errors so common? Put simply, much poor research arises because researchers feel compelled for career reasons to carry out research that they are ill equipped to perform, and nobody stops them. Regardless of whether a doctor intends to pursue a career in research, he or she is usually expected to carry out some research with the aim of publishing several papers. …. A common argument in favor of every doctor doing some research is that it provides useful experience and may help doctors to interpret the published research of others. Carrying out a sensible study, even on a small scale, is indeed useful, but carrying out an ill designed study in ignorance of scientific principles and getting it published surely teaches several undesirable lessons….Many people think that all you need to do statistics is a computer and appropriate software. This view is wrong even for analysis, but it certainly ignores the essential consideration of study design, the foundations on which research is built. Doctors need not be experts in statistics, but they should understand the principles of sound methods of research.”*

In a way, the text heralded the statements on improvement of methodological quality and reporting standards of medical research, such as the CONSORT statement (http://www.consort-statement.org/about-consort) pertaining to randomized controlled trials or the STROBE statement (https://www.strobe-statement.org/index.php?id=strobe-home) pertaining to observational studies. Twenty-five years later, warnings about “poor medical research” (2) are still issued. This is largely attributed to the fact that ”...*The curricula of most medical schools do not prioritize conduct and interpretation of medical research*” (2).

Mastering the art of (practical) medicine is a demanding task: it requires adoption of a myriad of facts and skills, ability of analytical and associative thinking, some common sense combined with trained intuition, and some talent – to result in sound clinical judgment and action. Our modest experience has been that quite commonly colleagues with a high level of expertise in their art do not adopt the same approach when it comes to conducting research; commonly, they tend to rely upon “common sense and intuition,” disregarding the fact that research methodology is a trade on its own that needs to be mastered. To illustrate some of the traps associated with relying (solely) on common sense and intuition, we here outline the main points of an imaginary research composed of elements of different reports that we have come across over the years.

A group of researchers have formulated a cream containing an otherwise well-known active substance that has been widely used topically as a compounded simple hydrogel in children to improve healing of superficial skin wounds, ie, scrapes/abrasions. The new formulation contains well-established inactive ingredients but is expected to yield an effect with only a single application. In an animal model, it showed an effect of accelerated healing. Scrapes/abrasions are minor ailments that heal spontaneously with only sporadic complications. However, they are extremely common in children and do interfere with some aspects of daily activities over a certain period of time: it might be practically useful if healing could be speeded-up by a simple single administration instead of repeated administrations over several days. A double-blind randomized placebo (simple hydrogel excipients)-controlled trial is planned in children 5-12 years of age as an initial evaluation of the new treatment. It aims primarily to evaluate safety, while assessment of efficacy is considered a secondary aim. The only known safety issue associated with repeated application of the existing formulation is a local transient hypersensitivity reaction manifested as light edema, redness, and itching of the surrounding skin. It is observed in around 2% of the treated children, and the researchers wish to assess whether the tested formulation is within this range. Hence, several visits are planned at which the wound would be inspected, and standardized high-quality photographs would be taken. Three researchers not involved in patient recruitment and follow-up would then, blinded to the treatment and patient, assess digital photos (3) to adjudicate whether local hypersensitivity/healing has occurred. To detect a possible accelerated healing two early time points were chosen at which some 20%-30% wounds spontaneously heal. The researchers sought advice and expressed their preferences: a) for career reasons they would prefer to complete this trial within 12 months; b) children with scrapes rarely seek professional medical attention – for both reasons, they considered it unfeasible to plan a trial that would need to include more than 80-90 children. The researchers were informed that: a) within these limits, it was impossible to reasonably demonstrate that the test formulation was “no worse” (ie, non-inferior) than placebo in respect to the incidence of hypersensitivity reaction, even if no cases were observed. However, with a sample of 80 children (randomized 1:1), a proportion of treated children with an event that is numerically identical or lower than that in the placebo group could be reasonably considered as an indicator that the test treatment was likely no worse than the existing one. Even if no events were to be observed in the placebo arm, with 0-1/40 treated children with an event, it would be more likely that the true incidence was ≤2% than that it was higher. If ≥2/40 cases were to be observed, it would be highly likely that the true incidence was >2%; b) assuming a time-averaged (time point 1, time point 2) proportion of “healers” in the placebo arm of 25%, such a sample size attains 80% power to detect a 23% higher absolute proportion of healers (ie, 48%) at two-sided alpha 0.1 – this corresponds to an odds ratio of 2.75: a fair indicator that the treatment indeed might accelerate healing to a relevant extent.

The trial started, but the patient enrollment was much slower than expected, and researchers decided to stop it after 18 patients had been randomized (9 to each arm). They reported no hypersensitivity reactions in either arm, but proceeded to an extensive evaluation of efficacy. The initial plan was changed *post hoc*, since the number of enrolled patients was too small to look at the proportion of “healers.” It appeared intuitive to grade the stage of “healing” at the two pre-defined time points. Several instruments have been developed to assess healing of chronic wounds, such as pressure ulcers or diabetic foot (4), but none specifically designed for simple superficial wounds (which is understandable, since such wounds are not nearly as relevant medical problem). It appeared intuitive that outcome assessment could be based on a simple judgement by the experienced investigators-pediatricians: in daily life, they inspect the wound and conclude that there is (i) “no healing (yet)”, (ii) “some healing,” or (iii) “obvious healing.” It also appeared intuitive that it would be practical to convert these judgments into numerical values for data analysis, eg, to values of 0, 1, or 2. Since judgment is subjective, it also appeared intuitive that each of the 3 raters should rate each wound at each time point 3 times.

Table 1 summarizes numerical scoring values for 3 imaginary patients in each treatment arm at the first and second assessment. It also illustrates a “problem” that emerged – what to do with all these zeros and ones for the analysis? Therefore, it appeared intuitive that they should somehow be “converted” into a more familiar format of “continuous-like” values: the researchers decided that all 3 scores by all 3 raters for one patient at one assessment should be summed-up and divided by 3 (3 raters) – to yield a “mean score” (across the 3 raters, for this patient at this particular assessment point). Alternatively, 3 ratings by one rater could be summed-up and divided by 3 – to yield a mean produced by this rater for this particular patient at this particular time point. Then, means for 3 raters could be summed-up and divided by 3 – to yield an overall mean to represent this particular patient’s healing status at this particular time point. Table 2 summarizes mean values for 3 patients in each treatment arm at the first and second assessment obtained by these procedures.

**Table 1:** Simulated rating scores of healing (three levels are possible: 0 = none; 1 = some, 2 = obvious) for 3 treated and 3 placebo patients at assessments 1 and 2, each rated 3 times by 3 raters*

PLACEBO – under code “A”					TREATED – under code “B”				
At assessment 1									
patient	scoring	rater 1	rater 2	rater 3	patient	scoring	rater 1	rater 2	rater 3
1	1st	0	1	1	1	1st	1	0	1
	2nd	0	1	1		2nd	0	1	0
	3rd	1	1	1		3rd	1	2	0
2	1st	1	1	0	2	1st	0	1	1
	2nd	0	0	1		2nd	2	1	1
	3rd	2	1	1		3rd	0	0	1
3	1st	1	0	0	3	1st	0	2	1
	2nd	1	1	1		2nd	1	1	1
	3rd	1	1	0		3rd	1	1	1
At assessment 2									
patient	scoring	rater 1	rater 2	rater 3	patient	scoring	rater 1	rater 2	rater 3
1	1st	1	1	1	1	1st	2	1	1
	2nd	1	1	1		2nd	1	1	1
	3rd	2	1	1		3rd	1	2	1
2	1st	2	0	1	2	1st	1	0	2
	2nd	1	2	0		2nd	1	1	1
	3rd	1	0	1		3rd	1	2	1
3	1st	0	2	1	3	1st	0	1	2
	2nd	2	2	1		2nd	0	1	1
	3rd	1	1	1		3rd	2	2	2

*Scores were generated by random sampling (function “sample” in R) with varying probabilities depending on time and treatment. Example: at the first assessment, the first patient on Placebo was rated 3 times by each of the 3 raters = a total of 9 scores. These 9 scores (range 0-2) were generated with probabilities: score 0 = 30%, score 1 = 65%, score 2 = 5%. Two further random samples (same probabilities) were generated for the second and third Placebo patient. The same was done for the 3 treated patients, but with a bit different probabilities: score 0 = 30%, score 1 = 60%, score 2 = 10%). At the second assessment, probabilities of “higher scores” were increased on the account of the “lower scores.” For Placebo patients: score 0 = 15%, score 1 = 60%, score 2 = 25%; for treated patients: score 0 = 10%, score 1 = 65% and score 2 = 25%.

**Table 2:** Mean scores obtained by averaging scores generated by each rater at 3 replications

Averaging of scores summed-up by rater						Averaging of mean scores by rater					
Placebo			Treated			Placebo				Treated	
patient		mean score	patient		mean score	patient			mean score	patient	mean score
**At assessment 1**											
1		2.33	1		2.00	1		0.78		1	0.66
2		2.33	2		2.33	2		0.78		2	0.78
3		2.00	3		3.00	3		0.66		3	1.00
**At assessment 2**											
1		3.33	1		3.66	1		1.11		1	1.22
2		2.66	2		3.33	2		0.89		2	1.11
3		3.66	3		3.66	3		1.22		3	1.22

The researchers then proceeded to evaluate treatment efficacy by using averages (9 treated and 9 placebo patients) in a repeated-measures ANOVA. Table 3 summarizes the results of this imaginary ANOVA (treatment and time point differences are identical regardless of which type of averaging is used). As expected, healing improved over time but there also appeared a “significant effect of treatment,” ie, treatment difference in “time-averaged score averages.” The researchers then extensively discussed the phenomenon of “significant acceleration of healing” (where the word “significant” was particularly stressed by the P value associated with the treatment difference depicted in Table 3) with the tested treatment.

**Table 3:** Summary of (the imaginary) repeated measures analysis of variance of averaged scores with treatment, time, and treatment*time interaction (df 1, F = 0.21, *P* = 0.655 for the interaction term)*

	Mean difference (95%CI)	df	t	P
Treatment vs placebo	0.09 (0.02-0.16)	1	2.77	0.013
2nd assessment vs 1st	0.35 (0.26-0.44)	1	8.70	<0.0001

*The (imaginary values) for 3 treated and 3 placebo patients in Table 2 were just triplicated to yield 9 patients per treatment arm.

Randomized controlled trial (RCT) is a clinical experiment with a wide range of methodological particulars – all research methods (eg, Western blotting, quantitative PCR, observational cohort studies, or any other) have their own “technical” particulars. The aim of an RCT (as a research method) is to provide an unbiased estimate of a (causal) treatment effect. This can only be achieved if its methodological principles are appreciated and followed. A number of books have been written on the concept and methodology of RCTs, in general and about a variety of its aspects (eg 5,6,), and the CONSORT website offers a “condensed tour” of the major (potential) issues (http://www.consort-statement.org/about-consort). In respect to the outlined imaginary research (which, however, contains elements taken from real trial reports, some of which were published in rather prestigious journals), we will focus on only a few points.

1. As a general concept, it may be acceptable to re-define/modify the outcomes and sample sizes in an ongoing trial (ie, “amend the protocol”). Such modification has to be approved by the same body that approved the initial trial proposal. One condition has to be met: this has to happen before trial outcomes are evaluated. We made no comments on whether this condition was met in the outlined imaginary trial, where researchers could have been particularly eager to demonstrate the functionality of their invention; the proposed changes have to adequately fit the purpose of the trial. In this respect, note: (i) the trial was reduced to around 20% of the initially planned sample size. This disabled any sensible safety assessment, which was originally depicted as a primary objective; (ii) the focus was completely shifted to efficacy. The initially defined outcome – proportion of “healers” – was straightforward, well defined, and practically relevant. The initially planned trial had a reasonable probability of “sensing” a certain potentially relevant treatment effect – had it existed. The “revised” trial version was reduced to a sample size not likely to detect even a huge effect. Moreover, rating was introduced. Rating scales are commonly used in many aspects of medical research, but in order to consider them reliable indicators of the process that is intended to be measured, they need to be validated, ie, assessed for content (logical) validity (that they do embrace different facets of the phenomenon intended for quantification), criterion and concurrent validity (extent to which the yielded results indeed measure the underlying process and extent of agreement with results obtained by a more accurate measurement means), sensitivity (ability to detect changes over time not due to measurement error), and inter- and intra-rater reliability (extent to which the same rater and different raters obtain similar results) (4). In the area of chronic wound healing, only some of the available instruments have been adequately validated (4). No clues were provided about any of the above properties for the scoring system consisting of 3 levels – “none,” “some,” and “obvious.”

2. No particular methodological knowledge –just some common sense – suffices to see that the approach with averaged scores is questionable. It might have been intuitive to opt for it, from a pragmatic standpoint, but it would not sustain even a simple superficial logical assessment. Only 3 numerical values have their descriptive counterparts: 0 (“none”), 1 (“some”), and 2 (“obvious”). What is a descriptive counterpart of a score of, eg, 2.33 or 3.66? Or of any non-integer score? What is the meaning of an average of 0.33 obtained for a patient who was scored: 0, 0, 1? Patient 1, in the placebo arm, at the first assessment (Table 1) was represented by a mean of 2.33 or 0.78 (Table 2) – where in fact, 7/9 assigned scores were 1, and two scores equaled 0. How does 2.33 or 0.78 reflect the healing status of this particular patient?

3. Each set of 3 numbers (by rater-by-patient-by time point) or 9 numbers (by patient-by time point) depicted in Table 1 has a certain dispersion – no measure of dispersion is provided along with the “averages” in Table 2, and the fact of this variability – within-rater (3 ratings on one occasion) or between raters – was completely disregarded. This resulted in an extremely small standard error of the estimated treatment-placebo difference in ANOVA – a difference of only 0.09 points yielded a t-value of 2.77 (t-value = difference/standard error of a difference; ie, standard error of this estimate was only 0.032) and an associated P-index of 0.013.

4. What is the meaning of a difference of 0.09 points of some arbitrary score?

5. The pitfalls of fascination with *P* values were addressed previously (7,8).

Already at this elementary level it is obvious that the reported analysis of efficacy is problematic: the numerical nature of the scoring process and the inter- and intra-rater variability were completely disregarded. Consequently, it does not make much sense, and the apparent effect could have easily been an artifact. In part, this is likely due to relying upon intuition. With the (very) modest methodological training that we get during medical education, it is more intuitive to us to search for “continuous-like variables”, ie, “something that could be analyzed by a t-test,” or ordinary least-squares regression-based techniques, such as repeated measures ANOVA. In this respect, it should be noted that the researchers in this imaginary trial did have an option to choose an outcome that would “fit this intuition.” Wound area reduction expressed as a percentage of the initial wound surface (by, eg, planimetry using digital photos) is a well established part of many of the chronic wound assessment instruments (4,9). For the setting of the outlined imaginary trial it would have been fully adequate. However, with the chosen outcome (scores of 0, 1, or 2), one could take into account the inter- and intra-rater variability only if its numeric nature was acknowledged – the outcome is a (repeatedly measured) ordered categorical variable. A way to analyze it (and estimate treatment effect) is to fit a cumulative logit model (10) to the probabilities of score 0, score 1, and score 2, taking into account each individual score produced by each rater for each patient. Table 4 summarizes results of such an analysis for the imaginary data depicted in Table 1 (but just replicated 3 times to mimic a situation with 9 patients per treatment arm). The (cumulative) odds ratio (ie, for higher-ordered vs lower-ordered scores) for assessment 2 vs 1 was 4.24 (2.71-6.64), clearly indicating healing over time, but there was no difference in time-averaged odds of higher-ordered scores in treated vs placebo patients (Table 4). Note that the estimated time-averaged probabilities generated in this analysis are in general agreement with the probabilities (with only slight differences in probabilities assigned to treatment and placebo) used to generate data in Table 1.

**Table 4:** Summary of the cumulative logit analysis: the method yields (cumulative) time-averaged (assessment 1, assessment 2) odds ratios (OR) of a higher-ordered vs lower-ordered score value (ie, 2 over 1 or 0; 1 over 0). Estimated time-averaged probabilities of individual scores in treated patients were 15.7% for 0, 84.3% for 1, and 15.7% for 2; while for the placebo patients the probabilities were 18.4% for 0, 81.6% for 1, and 13.3% for 2*

Effects	OR (95%CI)	df	Chi^2^	P
Treatment vs Placebo	1.21 (0.83-1.76)	1	1.02	0.313
Assessment 2 vs 1	4.24 (2.71-6.64)	1	36.1	<0.0001

*Data were analyzed using SAS 9.4 proc genmod with treatment, visit and treatment*visit interaction (df 1, Chi^2^ 0.01, *P* = 0.906 for the interaction term) as fixed effects; distribution multinomial; link = clogit; repeated subject = patient*rater(visit).

The *BMJ* editorial from 1994 (1) pointed out that “doctors need not be experts in statistics,” but that they should understand the principles of “sound methods of research.” In respect to the outlined imaginary research, this understanding (as opposed to “intuition”) is only slightly “statistical” (apart maybe from fitting the two models depicted in Table 3 and Table 4), and includes: a) understanding of the purpose and “technicalities” of an RCT; b) understanding of the importance of choosing a relevant outcome and an adequate quantification method; c) understanding of the properties of certain metrics and the importance of choosing appropriate methods of data analysis; d) understanding that “statistical analysis” is not about “searching for something/anything with a low P value”; e) understanding that a trial is a complex model, set up to test a specific hypothesis or a few hypotheses about a treatment, and that it needs to follow a pre-defined plan that should account for a number of its elements.
